# Middle meningeal artery embolization in chronic subdural hematoma: a paradigm shift?

**DOI:** 10.1007/s00330-026-12569-6

**Published:** 2026-06-10

**Authors:** Yan Li, Johannes Haubold, Natalie van Landeghem, Cornelius Deuschl, Ramazan Jabbarli, Philipp Dammann, Ulrich Sure, Isabel Wanke, Luisa Strack, Michael Forsting

**Affiliations:** 1 Department of Diagnostic and Interventional Radiology and Neuroradiology, University Hospital Essen, Essen, Germany; 2 Institute for Diagnostic and Interventional Radiology, Neuroradiology and Nuclear Medicine, University Hospital Knappschaftskrankenhaus Bochum, Ruhr University Bochum, Bochum, Germany; 3 Department of Neurosurgery and Spine Surgery, University Hospital Essen, Essen, Germany; 4 Swiss Neuroradiology Institute, Zürich, Switzerland; 5 Karl Landsteiner University of Health Sciences (KL) at the Krems Campus, Krems an der Donau, Austria

**Keywords:** MMA, Embolization, cSDH, MMAE, SDH

## Abstract

**Abstract:**

Chronic subdural hematoma (cSDH) is a common and growing neurosurgical condition, particularly in the elderly, with high recurrence rates following conventional surgical evacuation. Traditional management strategies primarily address mass effect through drainage but fail to target the underlying pathophysiological mechanisms responsible for hematoma persistence and recurrence. Increasing insights into the role of inflammation, angiogenesis, and fragile neovascular membranes supplied by the middle meningeal artery (MMA) have led to the development of MMA embolization as a targeted therapeutic approach. Over the past decade, MMA embolization has evolved from an experimental salvage technique to an evidence-based intervention. Early observational studies and meta-analyses demonstrated marked reductions in cSDH recurrence and reoperation rates compared with standard surgical management. More recently, large randomized controlled trials have provided consistent, high-level evidence confirming that MMA embolization, particularly as a surgical adjunct, lowers rates of hematoma recurrence, treatment failure, and serious adverse events. Consequently, contemporary consensus guidelines released in 2025 endorse MMA embolization as a class I recommended adjunct therapy for selected patients with nonemergent cSDH. Beyond its adjunctive role, MMA embolization is increasingly being explored as a standalone treatment option. This minimally invasive strategy holds particular promise for frail, elderly patients or those with significant comorbidities who are at high risk for surgical complications. Ongoing trials are aimed at refining patient selection, optimal intervention timing, and embolic techniques. In summary, current evidence supports a paradigm shift in cSDH management toward a pathophysiology-driven, preventive, and increasingly endovascular treatment model.

**Key Points:**

***Question*** *Does middle meningeal artery (MMA) embolization represent a paradigm shift in chronic subdural hematoma (cSDH) management?*

***Findings*** *Randomized trials show MMA embolization, as a surgical adjunct, reduces recurrence and treatment failure in cSDH, earning a Class I guideline recommendation.*

***Clinical relevance*** *MMA embolization shifts cSDH management from reactive surgical drainage to a proactive strategy targeting the pathologic neomembranes. This pathophysiology-based approach prevents hematoma recurrence, improving long-term outcomes and establishing a new treatment paradigm.*

## Introduction

Chronic subdural hematoma (cSDH) is among the most common cranial pathologies in older adults, with an incidence of approximately 8–17 per 100,000 persons per year [1–3]. The incidence of cSDH rises sharply with age, with reported rates escalating from 76.5 per 100,000 persons per year in the 70–79 age bracket to 127 in individuals over 80 [4]. With an estimated 60,000 cases, cSDH is projected to become the most common adult cranial neurosurgical condition in the U.S. by 2030 [5]. Clinically, patients often present with headaches, cognitive decline, gait disturbance, or focal neurological deficits. The 30-day mortality rate partly or fully attributable to cSDH has been reported to be as high as 9.4% [2]. While small and asymptomatic cSDHs may be observed conservatively with serial imaging, symptomatic or large cSDHs are managed surgically, with twist-drill or burr-hole craniostomy being the first-line approaches [6–10].

A major challenge in cSDH management is the high recurrence rate, ranging from 10% to 20% after initial surgical evacuation [11–14]. Known risk factors for recurrence include advanced age, significant brain atrophy, bilateral hematomas, anticoagulation/antiplatelet therapy, and certain radiological hematoma types (e.g., mixed- or high-density composition, separated and layered internal architecture, and midline shift greater than 5 mm) [15–18]. Each recurrence not only necessitates hospital readmission and repeat surgery with increased patient morbidity, but also generates additional costs that may exceed those of initial treatment, with one study reporting up to 2-fold higher expenses due to prolonged hospital stays and more frequent outpatient follow-ups compared to the index procedure [19]. Therefore, management of cSDH imposes a growing financial burden on healthcare systems worldwide, driven primarily by its increasing incidence in aging populations and the substantial costs associated with treating its high recurrence rate.

## Rationale for middle meningeal artery embolization

In light of the recurrence problem, there has been growing interest in therapies targeting the underlying cSDH pathophysiology rather than only repeatedly draining recurrent fluid accumulation. Typically, cSDH develops weeks after minor head trauma, driven by inflammation and angiogenesis within the subdural space that leads to fragile neocapillary formation in an outer dural membrane [20, 21]. These leaky vessels, mainly supplied by branches of the middle meningeal artery (MMA), might cause recurrent bleeding and fluid exudation that sustain the hematoma cycle [22, 23]. Angiographic studies corroborate that the ipsilateral MMA is often enlarged where cSDH occurs, with a diffuse network of abnormal capillaries to supply the hematoma membrane [24]. These histopathological and angiographic findings led to the hypothesis that MMA occlusion could devascularize the hematoma membranes, halting the cycle of microhemorrhages and exudation and thereby stabilizing or even accelerating the resolution of the hematoma [25, 26].

The first reported case of MMA embolization for cSDH was published by Mandai et al in 2000 [27]. In that case, a 59-year-old man with coagulopathy caused by liver cirrhosis had a refractory, recurrent cSDH despite multiple surgeries. Embolization of the ipsilateral MMA with particles (polyvinyl alcohol, PVA) was performed, resulting in near-complete hematoma resolution at 7-month follow-up and no further recurrence. This landmark case demonstrated the feasibility of MMA embolization for preventing cSDH recurrence. Over the next two decades, numerous case reports and small series described MMA embolization with encouraging results in patients who either failed conventional management or were poor surgical candidates [28–30]. Subsequently, larger prospective cohort studies systematically reported promising outcomes with MMA embolization. A study by Link et al in 2018 observed only an 8.9% recurrence after MMA embolization in 60 cases [31]. Another study reported an extremely low treatment failure rate of 1.4% in 72 cSDH patients undergoing MMA embolization compared to 27% of 469 patients under conventional treatment [32]. These early non-randomized studies suggested that MMA embolization could significantly reduce the need for reoperations and perhaps even serve as a primary therapy option for cSDH management.

Later on, a meta-analysis in 2018, pooling three double-arm studies comparing embolization and conventional surgery and 6 single-arm with case series, found that the embolization group had a composite recurrence rate of only 2.1%, significantly lower than the 27% recurrence by conventional management [33]. Another meta-analysis of 20 studies including 1416 patients in 2021 confirmed significantly lower rates of cSDH recurrence (OR = 0.15 [95% CI 0.03 to 0.75], *p* = 0.02) and surgical rescue (OR = 0.21 [0.07 to 0.58], *p* = 0.003) by MMA embolization compared to conventional management [34].

## Current clinical evidence for MMA embolization

Between 2024 and 2025, the paradigm shifted with the completion of several high-quality randomized controlled trials (RCTs) (Table 1), which for the first time provide prospective evidence on MMA embolization efficacy.

**Table 1:** Latest landmark randomized controlled trials from 2024 to 2025

Trial	Design	Sample (*N*)	Primary endpoint	Key outcomes	Clinical takeaway
EMBOLISE	Adjunct to surgery burr-hole craniostomy vs. surgery alone	400	Recurrence requiring surgery at 90 days	4.1% (MMAE) vs. 11.3% (control)	Devascularization with EVOH (Onyx) reduces recurrence by ~3x.
MAGIC-MT	Standalone or adjunct vs. standard care	722	Symptomatic recurrence/progression at 90 days	6.7% (MMAE) vs. 9.9% (control) (*p* = 0.10)	Modest radiographic benefit; significant reduction in serious adverse events (*p* = 0.02).
STEM	Standalone or adjunct vs. standard care	310	Composite failure (residual > 10 mm, surgery, death)	16% (MMAE) vs. 36% (control) (*p* = 0.001)	Highly significant reduction in treatment failure using liquid embolic (Squid).
EMPROTECT	Adjunct to surgery vs. surgery alone	342	Recurrence at 6 months	14.8% (MMAE) vs. 21.0% (control)	Use of particles (300–500 μm) showed a favorable trend but no statistical significance.
MEMBRANE	Standalone or adjunct vs. standard care	376	Composite failure at 6 months	8.5% (adjunct) vs. 20.2% (surgery only)	Significant benefit for n-BCA glue, especially in patients aged > 75.

*MMAE* middle meningeal artery embolization, *EVOH* ethylene-vinyl alcohol copolymer, *n-BCA* n-butyl cyanoacrylate

### EMBOLISE (2024)

This multicenter RCT in the U.S. and Europe enrolled 400 patients with symptomatic subacute/chronic SDH requiring surgery [35]. Patients were randomized to standard neurosurgical treatment (burr-hole drainage) vs. surgery plus adjunctive MMA embolization with Onyx (ethylene-vinyl alcohol copolymer, EVOH). At 90-day follow-up, the addition of MMA embolization led to a three-fold reduction in hematoma recurrence requiring repeat surgery: only 4.1% of the embolization group versus 11.3% of the surgery-only group. Serious adverse events related to embolization were uncommon (approximately 2%).

### MAGIC-MT (2024)

Conducted in China, this was the largest RCT, enrolling 722 patients across 26 centers [36]. Patients were then randomized 1:1 to receive MMA embolization with Onyx or no embolization, stratified by whether they underwent burr-hole drainage or not. The primary endpoint—symptomatic hematoma recurrence or progression by 90 days—was slightly lower in the embolization group (6.7% vs. 9.9% in controls), but this difference (3.2%) did not reach statistical significance. Notably, however, serious adverse events, including deaths, occurred in only 6.7% of patients with embolization compared to 11.6% with standard care (*p* = 0.02). This trial suggests that MMA embolization may be a safe alternative or adjunct, but when surgery is already performed in most patients, the incremental benefit in preventing radiographic recurrence might be modest.

### STEM (2025)

This international RCT broadened the scope by including patients for whom initial management could be surgical or nonsurgical [37]. 310 patients with symptomatic cSDH were randomized to standard management (burr-hole drainage or observation/medical management) versus standard management plus previous MMA embolization with Squid (ethylene-vinyl alcohol copolymer, EVOH). The composite primary outcome (treatment failure within 180 days, defined as significant residual/recurrent hematoma > 10 mm, need for rescue surgery, or major neurological morbidity) occurred in 16% of the embolization group versus 36% of the control group. This 60% relative risk reduction favoring MMA embolization was highly significant (OR = 0.36, *p* = 0.001). The STEM investigators concluded that adjunctive MMA embolization markedly lowers cSDH treatment failure without adding short-term risk.

### EMPROTECT (2025)

This was a French trial focusing specifically on post-surgical recurrence prevention. 342 patients underwent burr-hole evacuation and were randomized to adjunctive MMA embolization versus no embolization [38]. Uniquely, this trial employed particles (300–500 μm, Embosphere) as the embolic agent rather than liquid embolics. At 6 months, cSDH recurrence occurred in 14.8% of the embolization group versus 21.0% of the control group—a favorable trend but not statistically significant (aOR = 0.64, 95% CI 0.36–1.14). Thus, EMPROTECT did not demonstrate a clear benefit to MMA embolization. The investigators noted that the relatively large particles might not penetrate distal capillaries sufficiently, potentially explaining the lack of effect.

### MEMBRANE (ongoing)

The MEMBRANE study is a U.S. randomized clinical trial evaluating the efficacy of a liquid cyanoacrylate glue (n-BCA) for MMA embolization, either adjunctive to surgery or as nonsurgical standard care [39]. Within the surgical arm, patients were randomized to surgery plus MMA embolization (*n* = 133) or surgery alone (*n* = 132). While full results are not yet published, preliminary data have been promising. At 6 months, residual or reaccumulated cSDH (> 10 mm) or the need for rescue surgery was 8.5% in the surgery plus MMA embolization group, 20.2% in the surgery-alone group, 20.2% in the nonsurgical plus MMAE group, and 27.0% in the nonsurgical-alone group. The trial demonstrated significant benefits of MMA embolization adjunct with surgery at 6 months, a favorable trend with MMA embolization in nonsurgical management and a significant treatment effect for MMA embolization in patients aged ≥ 75 years undergoing surgery [40].

A thorough understanding of the neurovascular anatomy relevant to MMA embolization is essential for safe and effective procedural performance. Knowledge of potential anastomotic channels of MMA, identification of cranial nerve supply, and appropriate selection of embolic material are critical to avoid neurologic complications [41, 42]. While individual trial data show some variation in reported complication rates, the collective evidence from these large RCTs demonstrates that MMA embolization has a favorable and consistent safety profile. A recent meta-analysis of the published four trials (EMBOLISE, STEM, MAGIC-MT, and EMPROTECT), encompassing 1774 patients, confirmed that MMA embolization is not associated with an increased risk of serious adverse events (pooled risk ratio (RR) = 0.91, 95% CI: 0.71–1.16, *p* = 0.45) [43]. Another meta-analysis similarly found no significant increase in serious adverse events (RR = 0.92, *p* = 0.47) or mortality (RR = 0.94, *p* = 0.85) compared to standard care [44].

Based on recent RCT evidence, the Society of Vascular and Interventional Neurology (SVIN) released a 2025 consensus statement giving a class 1 recommendation for MMA embolization as an adjunct to standard care for symptomatic, nonemergent, and nonrecurrent chronic subdural hematomas [40]. The guideline also recommends using liquid embolic agents (e.g., Onyx, Squid, or n-BCA) for more controlled and sustained vessel occlusion (class 1).

Thus, MMA embolization has evolved from an experimental approach in 2000 to an established, evidence-based therapeutic strategy, supported by high-quality RCTs. Beyond reducing long-term recurrence and reoperation, it also provides rapid clinical benefits, including prompt relief of chronic headaches, which enhances patient satisfaction due to its minimally invasive nature and potential to avoid open surgery [45].

## Cost-effectiveness in the context of MMA embolization

Cost-effectiveness is a critical consideration in cSDH management alongside therapeutic efficacy. A propensity-score matched analysis of 33 embolization and 33 surgical cases found that although procedural costs were substantially higher for embolization ($38,255 ± $11,859 vs. $11,206 ± $7888; *p* < 0.001), total index hospitalization costs were not significantly different ($71,569 ± $37,813 vs. $60,598 ± $61,315; *p* = 0.385). This cost equivalence resulted from higher procedural and embolic agent costs being offset by reduced intensive care, imaging, and postoperative expenses (e.g., wound care, anesthesia) associated with open surgery [46]. A 5-year U.S. database analysis (2017–2022) found that combining surgical drainage with MMA embolization is more cost-effective than surgery alone over a 6-month period. Although the upfront cost of the combined procedure was higher ($74,568 vs. $39,658; *p* = 0.003), the total median healthcare payments at 6 months were significantly lower for the embolization group ($7300 vs. $11,494; *p* = 0.0017). This long-term savings is attributed to reduced complication and infection rates [47]. A propensity-adjusted analysis of 170 patients demonstrated significantly lower total 1-year hospital costs for patients treated with MMA embolization compared to traditional surgery (mean difference −$32,776; 95% CI −$52,766 to −$12,787; *p* < 0.001). The substantial long-term cost savings in the embolization group were directly linked to a markedly lower retreatment rate (4% vs. 16% in the surgical cohort), where the need for repeated procedures was a key driver of higher expenses [48].

Cost-effectiveness analyses of the Nationwide Readmissions Database (9532 patients) demonstrated that MMA embolization was associated with an additional 1.3 quality-adjusted life years over a lifetime (approximately 16 months of perfect health), compared with conventional treatment. The incremental cost-effectiveness ratio was $15,199.8 per quality-adjusted life year, well below the willingness-to-pay threshold of $50,000–100,000 in the U.S. These findings further support a paradigm shift toward MMA embolization in cSDH treatment [49].

Based on the current literature, MMA embolization, despite higher initial procedural costs for specialized equipment and embolic agents, proves to be a cost-effective long-term strategy. This economic advantage is primarily driven by its efficacy in significantly reducing rates of hematoma recurrence and the need for rescue surgery.

## Future perspectives

The integration of MMA embolization into cSDH management is rapidly evolving. However, its optimal application is defined by several critical knowledge gaps that are the focus of intense contemporary research. Addressing these questions is essential to refine patient selection, maximize therapeutic efficacy, and solidify its role within a precision-based treatment paradigm.

### Optimal patient selection and embolization timing

Several ongoing RCTs are centrally focused on resolving two key clinical questions: which patients benefit most from MMA embolization and when the procedure should be performed. For instance, the STORMM trial systematically evaluates embolization efficacy through four study arms: surgery alone, surgery plus adjuvant embolization within 72 h, embolization alone, and conservative management. This design covers four clinical scenarios and directly tests the hypothesis that early postoperative embolization disrupts the pathophysiological cycle driving recurrence, thereby defining a critical therapeutic window [50]. Concurrently, the CHESS trial investigates a paradigm shift by comparing standalone MMAE to conventional surgical evacuation in patients with symptomatic cSDH not yet mandating emergency surgery. Its goal is to establish MMA embolization as a non-inferior, minimally invasive first-line therapy, potentially sparing frail, elderly patients the risks of open surgery [51]. Further, expert consensus of SVIN highlights a critical knowledge gap and calls for future RCTs to evaluate embolization as an adjunct in neurologically unstable, emergent presentations [40, 52], an area not addressed by prior major trials.

### Bilateral versus unilateral MMA embolization and embolic agent selection

While major RCTs have established the efficacy of unilateral MMA embolization, the bilateral approach has been increasingly adopted in clinical practice. However, comparative evidence remains contradictory and stems primarily from non-randomized data [53, 54]. Preliminary case series indicate potential benefit for a bilateral embolization, with one study reporting a recurrence rate of 6% for bilateral procedures (*n* = 83) versus 12.7% for unilateral ones (*n* = 55) (*p* = 0.087) [55]. In contrast, another study comparing 113 unilateral with 54 bilateral embolization cases found unilateral embolization was associated with a significantly higher non-recurrence rate (96.91% vs. 84.78%, *p* < 0.01) and a lower complication rate (5.41% vs. 18.52%, *p* < 0.01) over a median follow-up of 71.5 days [56]. This clinical equipoise underscores the need for prospective controlled studies to determine if bilateral embolization that requires longer interventional time and potentially carries higher periprocedural complication rates should be generally preferred for an optimal outcome.

The selection of embolic materials remains another area of clinical equipoise, with particles, liquid embolics (EVOH copolymers or n-BCA glues), and coils each presenting not only distinct technical profiles but also great differential cost [57–60]. While EVOH agents are widely preferred for their controlled penetration and durable occlusion, direct comparative evidence is scarce and contradictory [61–64]. The choice of embolic agent currently rests on operator experience, institutional protocol, and inference from retrospective data.

### Grading systems and predictive models

The expanding role of MMA embolization also necessitates the development of validated predictive models to guide patient selection. An ideal prognostic tool would integrate clinical, anatomical, and radiological predictors into a standardized scoring system to identify patients most likely to achieve clinical and radiographic success. These include specific radiological and anatomical features, such as membrane enhancement [65–68], hematoma density and subtypes according to different internal architecture on CT (e.g., the Nakaguchi classification) [69–72], and MMA angio-architecture characteristics (e.g., vessel caliber) [73]. Furthermore, clinical factors such as the use of anticoagulant or antiplatelet therapy were reported as potentially negative modulators of outcome [74–76]. The synthesis of diverse predictive markers into a validated grading system seems to be the next essential step in cSDH management. This would enable a paradigm shift from a reactive model of surgical evacuation to a proactive, precision-based strategy of targeted devascularization in better-selected patients.

### Imaging and quality-of-life follow-ups after MMA embolization

**Fig. 1:**
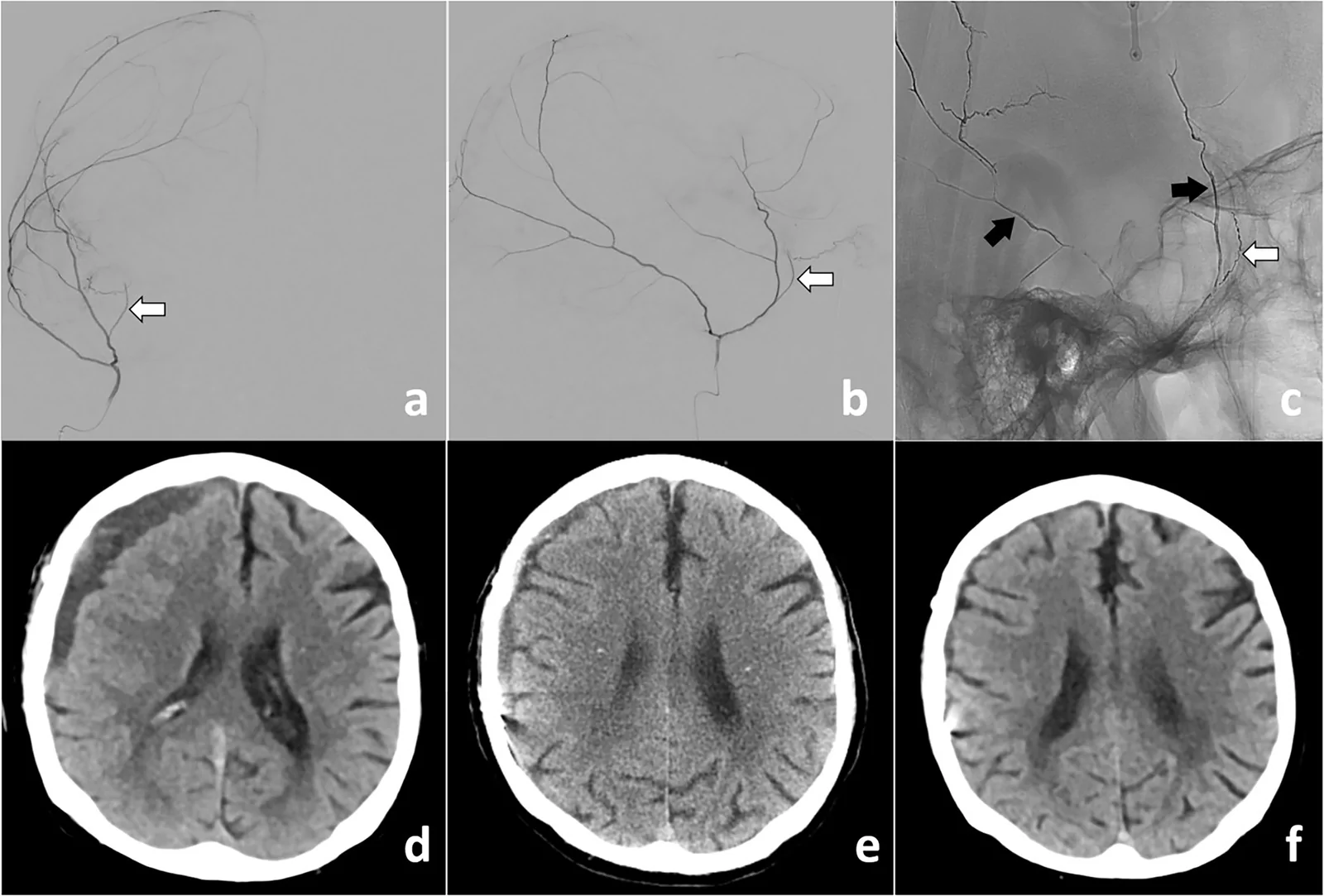
Middle meningeal artery embolization and follow-up in an 88-year-old male with recurrent chronic subdural hematoma. The patient underwent MMA embolization for a right-sided cSDH recurrence after initial surgical evacuation. **a**, **b** Superselective digital subtraction angiography (DSA) in frontal and lateral projections shows enlargement of the right MMA and its sphenoidal orbital branch (white arrow). **c** To prevent orbital complications, the orbital branch was coil-embolized (white arrow), followed by embolization of the frontal and parietal branches with liquid embolic agent (Onyx, black arrow). **d**–**f** Axial CT images demonstrate the hematoma volume post-procedure (**d**), showing partial resolution at 3-month follow-up (**e**), and complete resolution at 8-month follow-up (**f**)

Unlike surgical evacuation, which provides immediate decompression, MMA embolization relies on a gradual, physiological resorption process. Consequently, the imaging follow-up schedule should track this evolution over months rather than days (Fig. 1) and remains undetermined. In most studies, imaging follow-up is performed 1 day post-intervention (surgical or embolization) and subsequently at 1, 3, and 6 months, then subsequent scans only for new or persistent symptoms [77]. As more data accumulate, guidelines can be refined regarding how often to perform imaging after embolization and for how long to follow patients clinically. The goal is to detect any rare late recurrence while avoiding unnecessary radiation and anxiety. Initial 1-year follow-ups from trials show sustained results [78]. As more long-term data emerge, we will understand whether MMA embolization merely delays recurrence or truly cures the condition in most cases.

Another important aspect is the health-related quality-of-life assessment. Although the MAGIC-MT trial reported no significant difference in health status between the embolization and control groups at 90 days (mean EQL-5D-5L score 0.95 vs. 0.94, as measured by the EuroQoL Group 5-Dimension 5-Level Self-Report Questionnaire), it remains unclear whether this equivalence persists over time, particularly given the higher recurrence rate of cSDH in the neurosurgical group [36]. The forthcoming SWEMMA trial may offer additional insights at 12 months [79]; however, more extensive long-term data are still needed, as the full impact of embolization on functional status and well-being may become apparent years after the procedure.

## Conclusion

Middle meningeal artery embolization has emerged as a transformative adjunct and potential alternative to conventional surgical management of chronic subdural hematoma. Latest high-quality randomized controlled trials now demonstrate its ability to significantly reduce recurrence and treatment failure while maintaining a favorable safety profile, particularly when liquid embolic agents are used. Although uncertainties remain regarding optimal patient selection, embolic material choice, and embolization timing as well as technique, ongoing trials and consensus guidelines increasingly support its integration into standard care. As evidence matures, MMA embolization is poised to shift cSDH management from reactive surgical evacuation toward a proactive, pathophysiology-driven, precision-based strategy.

## Abbreviations

CHESS: Chronic subdural hematoma embolization vs. surgery study
CI: Confidence interval
cSDH: Chronic subdural hematoma
CT: Computed tomography
EMBOLISE: Embolization of the middle meningeal artery with Onyx liquid embolic system in the treatment of subacute and chronic subdural hematoma
EMPROTECT: Embolization of the middle meningeal artery with particles for the prevention of recurrence of chronic subdural hematoma
EVOH: Ethylene-vinyl alcohol copolymer
MAGIC-MT: Middle meningeal artery embolization for chronic subdural hematoma management trial
MEMBRANE: Middle meningeal artery embolization for the treatment of chronic subdural hematomas
MMA: Middle meningeal artery
MMAE: Middle meningeal artery embolization
n-BCA: n-Butyl cyanoacrylate
OR: Odds ratio
RCT: Randomized controlled trial
SDH: Subdural hematoma
STEM: The SQUID trial for the embolization of the middle meningeal artery for treatment of chronic subdural hematoma
STORMM: Subdural hematoma trial of MMA embolization
SVIN: Society of Vascular and Interventional Neurology
U.S.: United States
